# Seeing Food Through Young Children’s Eyes: Children’s Representations of Parental Feeding Strategies and Food Choice Reasoning

**DOI:** 10.3390/children13030347

**Published:** 2026-02-27

**Authors:** Irith Freedman, Anat Gesser-Edelsburg, Billie Eilam

**Affiliations:** 1 Department of Interdisciplinary Social Sciences, The Max Stern Yezreel Valley College, Emek Yezreel 1930600, Israel; 2 Department of Human Services, The Max Stern Yezreel Valley College, Emek Yezreel 1930600, Israel; 3 Department of Humanities and Arts, Technion—Israel Institute of Technology, Haifa 3200003, Israel; 4 School of Public Health, Faculty of Social Welfare and Health Sciences, University of Haifa, Haifa 3498838, Israel; ageser@univ.haifa.ac.il; 5 Faculty of Education, University of Haifa, Haifa 3498838, Israel; beilam@edu.haifa.ac.il

**Keywords:** early childhood, nutrition education, child agency, parental feeding practices, qualitative research

## Abstract

**Highlights:**

**What are the main findings?**

- Young children portray parents as emphasizing health, control, and negotiation when guiding food intake, yet their own food choices are primarily driven by personal preference.
- For most children, representations of parental feeding strategies do not align with the considerations guiding their immediate food-choice decisions.

**What are the implications of the main findings?**

- Food-related socialization in early childhood is contextual and interpretive, with children actively selecting when and how parental messages inform their choices.
- Nutrition education and health promotion efforts may benefit from explicitly engaging children’s agency and preferences rather than relying solely on adult-directed health messaging.

**Abstract:**

**Background/Objectives**: Research on children’s eating has primarily focused on parental feeding practices and dietary outcomes, with less attention to how young children themselves understand parental food-related messages and relate them to their own food choices. Recognizing children as active participants in food socialization, this study aimed to examine preschool children’s representations of parental feeding strategies alongside their expressed food-choice considerations. **Methods**: A qualitative, exploratory, multi-method design was employed within a constructivist framework. Forty kindergarten children aged 4 years 10 months to 5 years 8 months participated in individual, play-based sessions conducted in familiar educational settings. Data were generated using two complementary tools: a doll role-play task eliciting children’s representations of parental feeding strategies and a simulated grocery shopping task eliciting food-choice considerations. All sessions were audio-recorded, transcribed verbatim, and analyzed using reflexive thematic analysis. **Results**: During role-play, children frequently portrayed parents as emphasizing health-related arguments, control, and negotiation when guiding food intake. Less frequently, they represented strategies such as encouragement to try, deception, or references to body weight. In contrast, during the food-choice task, children’s selections were primarily guided by personal preference, with health considerations mentioned less often. For most participants, the feeding strategies attributed to parents did not closely align with the considerations guiding their own food choices. **Conclusions**: The findings highlight young children’s active and selective engagement with parental feeding discourse and underscore the contextual nature of food-related meaning-making in early childhood. Rather than reflecting a straightforward transmission of parental messages, children’s food choices appear shaped by situational affordances and perceived autonomy, supporting child-centered approaches to nutrition education and health promotion.

## 1. Introduction

For the past several decades, efforts to improve children’s diets have focused primarily on promoting healthy nutrition and preventing childhood overweight and obesity—conditions that have increased worldwide and are associated with both immediate and long-term health consequences [1,2,3]. Public health initiatives have therefore emphasized increasing children’s intake of nutritious foods, such as fruits and vegetables, while reducing consumption of energy-dense, nutrient-poor foods, including sweets and snacks [4]. More recently, child healthcare and health education research has increasingly recognized that achieving these goals requires not only attention to dietary outcomes, but also a deeper understanding of how children themselves experience, interpret, and respond to food-related messages in their everyday lives [5,6,7].

Parents play a central role in efforts to support children’s balanced diets and healthy eating habits, employing a wide range of feeding strategies and practices [8,9]. Extensive research has examined parents’ feeding approaches and how these are communicated to, perceived by, and responded to by children, highlighting different feeding styles, attitudes, and behaviors [10,11,12,13,14]. These include parental modeling and various forms of behavioral guidance, health-related messages about food [15,16], and the use of rewards or punishments to influence children’s eating [12,17,18]. Qualitative examinations of everyday parent–child feeding interactions further suggest that these approaches tend to cluster into broader patterns of practice, ranging from more directive and controlling strategies (such as pressure, restriction, or the use of threats and incentives) through structured forms of guidance (including routines, rules, and monitored choice) to more autonomy-supportive approaches that rely on reasoning, negotiation, encouragement, and children’s involvement in food preparation [19]. Importantly, parents appear to adapt these strategies in relation to the perceived characteristics and needs of each child, adjusting portion sizes, limiting specific foods, or encouraging intake according to factors such as age, weight status, or pickiness [20]. Evidence from community-based contexts further indicates that institutional guidance, such as that provided by childcare or healthcare settings, may reinforce either structured or more restrictive feeding approaches, shaping how parents enact these strategies in everyday life [21]. While these strategies are well documented, their influence on children’s eating is not uniform, and children’s responses to parental feeding practices vary considerably across contexts and developmental stages. Importantly, much of this research has focused on parental practices from the adult perspective (e.g., [14,15]), with comparatively less attention to how children themselves understand and interpret these strategies.

Although parents are a central influence, children’s food-related attitudes and behaviors develop within broader ecological contexts. Drawing on ecological systems theory [22,23], parents’ feeding practices can be understood as one component within a network of interacting social, cultural, and environmental influences. Prominent factors repeatedly shown to shape children’s food preferences include media messages and food advertising directed at children [24,25], genetic and environmental conditions [26], and the influence of institutional actors such as education and health providers [21,27]. Within this multi-layered environment, children are not merely passive recipients of food-related messages but active participants in how such messages are interpreted, negotiated, and, at times, resisted. This dynamic is reflected, for example, in research documenting negative associations between highly controlling parental behaviors and children’s food consumption. [28,29,30,31]. At the same time, children’s own preferences, tastes, and established habits constitute central determinants of their food-related behaviors [32,33].

Recent integrative reviews in child health further emphasize that children’s eating behaviors emerge through ongoing, bidirectional interactions between caregiver feeding practices, food environments, and children’s own characteristics and responses, underscoring the importance of child-centered approaches to food socialization research [34]. Children’s active role in their own socialization has been recognized since the 1970s, with early accounts describing socialization as a process of selective internalization of sociocultural information derived from multiple sources, including parents [35]. This selective process is evident in food-related socialization as well [36], throughout which children actively participate in food choices and often express a desire to exercise control over what they eat [37,38,39]. Children also influence food-related practices within the family, including meal planning [37] and grocery shopping decisions [40]. Moreover, food-related attitudes constructed in early childhood may shape later beliefs and behaviors in adulthood [41,42].

Despite extensive research on parental feeding strategies and growing recognition of children’s involvement in their own food socialization, relatively little is known about how young children internalize and make sense of parental feeding discourse within their developing understandings of food. Existing work has largely examined parents’ reported practices or children’s observable eating behaviors, with less attention to how parental messages are taken up and reflected in children’s representations and expressed reasoning. To address this gap, the present study adopts a child-centered perspective that foregrounds children’s voices and interpretive processes. Specifically, it aims to explore young children’s portrayals of parental feeding discourse alongside their articulated food-choice reasoning and, in turn, to consider possible points of convergence and divergence between these two domains.

## 2. Materials and Methods

This study employed a qualitative, exploratory, multi-method design grounded in a constructivist epistemological framework [43]. The design aimed to elicit young children’s self-expressed interpretations of parental feeding strategies and their own food-choice considerations through age-appropriate, play-based methods. Consistent with child-centered qualitative inquiry, the study conceptualizes children as active participants in shaping, expressing, and negotiating their own knowledge; accordingly, children’s verbal expressions, role-play enactments, and choices were treated as meaningful contributions that reflect their ongoing sense-making processes within their social and cultural contexts. To support such expression, the design integrated two complementary qualitative elicitation tools, doll role-play and a simulated food-choice task, reflecting broader methodological insights that young children’s tacit and explicit knowledge is more effectively accessed through interactive and symbolic tools than through direct questioning alone [44].

### 2.1. Ethical Considerations

The study was ethically approved by the Chief Scientist of the Israeli Ministry of Education (Approval No. 8278/266). Written informed consent was obtained from parents prior to children’s participation. Children received an age-appropriate explanation of the study and were asked for their verbal assent before participation. Participation was voluntary, and children were informed that they could withdraw at any time.

In line with Article 12 of the United Nations Convention on the Rights of the Child [45], which affirms children’s right to express their views in matters affecting them, the study respected children’s voluntary participation, and all children whose parents provided written consent and who themselves expressed a willingness to participate (*N* = 40) were included in the study. Data collection took place in familiar kindergarten settings, and care was taken to ensure children’s comfort and well-being throughout the sessions. The researcher monitored children’s engagement and responded sensitively to signs of fatigue or discomfort. All data were anonymized and stored securely, with access restricted to the research team.

### 2.2. Researcher Positionality

The research team comprised scholars in education, health communication, and cognitive development, all with prior experience conducting qualitative research with young children. The first author was responsible for facilitating the play-based activities, engaging with children during the role-play and food-choice simulation tasks and eliciting their explanations and reflections, as well as leading the qualitative data analysis. This close interaction enabled attentiveness to children’s communicative styles, engagement levels, and meaning-making processes during data generation, supporting rich and contextually grounded interpretations.

This positioning entailed potential risks of interpretive influence, particularly given the asymmetrical power relations [46,47] between an adult researcher and young child participants, as well as the researchers’ role in shaping the structure and prompts of the data-elicitation tools. To address these risks, reflexive practices were employed throughout the study, including analytic memo-writing during data analysis, ongoing critical reflection on the researchers’ assumptions regarding children’s food-related knowledge and agency, and peer analytic discussions among the authors during theme construction [46,48].

Transparent acknowledgment of the researchers’ roles and their potential influence on data generation and interpretation was treated as a core methodological principle. By explicitly engaging in reflexive and collaborative analytic practices, the study aimed to enhance the credibility, rigor, and trustworthiness of its qualitative findings [48].

### 2.3. Participants and Recruitment

Participants were forty kindergarten children (21 boys, 19 girls), aged between 4 years 10 months and 5 years 8 months, recruited from five kindergartens in northern Israel. Of these, *n* = 12 and *n* = 6 children attended two kindergartens located in areas classified by the municipal authority for tax purposes as having a high socio-economic status (SES) (total *n* = 18), while *n* = 7, *n* = 7, and *n* = 8 children attended three kindergartens located in areas classified as low-medium SES (total *n* = 22). Although SES was considered in the initial stages of the analytic process, no meaningful SES-related differences were observed. In line with the qualitative aims of the study, analyses were therefore not stratified by SES. Following ethical approval from the Ministry of Education, the research team contacted the municipal authorities’ early childhood education departments, or equivalent offices responsible for kindergarten oversight, to obtain institutional cooperation and to facilitate contact with relevant lead teachers. Subsequently, six kindergarten head teachers were approached and provided with a detailed explanation of the study’s aims and procedures, and five of them agreed to facilitate recruitment by distributing information and consent forms to parents. Following the distribution of information sheets and the acquisition of parental consent, as outlined in Section 2.1, parents who wished their child to participate returned signed consent forms to the kindergarten teacher. Children whose parents provided written consent were then approached individually and invited to participate in the study. Forty children who indicated their willingness to participate were included in the study.

### 2.4. Data Generation Tools and Procedure

**Figure 1:**
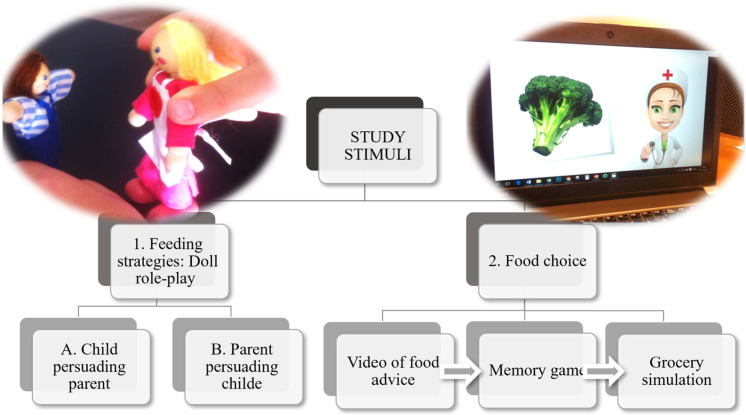
Diagram of study stimuli administration.

Data collection took place in a quiet corner of the kindergarten, where there were no interruptions or spectators. Each session was conducted individually with one child, and at the beginning of the session, the researcher confirmed each child’s voluntary assent and explained that there were no right or wrong answers, that she was interested in everything the child had to say, and that their ideas could help adults learn more about the topic. The researcher also asked for the child’s permission to audio-record the session and demonstrated how the recording device worked by making a brief test recording and playing it back to the child. Two main stimuli were administered in a single individual session: (1) a doll role-play task and (2) a food-choice elicitation sequence comprising an animated video, a memory game, and a grocery shopping simulation (see Figure 1). Each individual session lasted approximately 15–20 min.

#### 2.4.1. Doll Role-Play

The first stimulus, a doll role-play task, was designed to elicit children’s verbal expressions reflecting their processed and internalized parental feeding-related strategies. Doll role-play was selected as a data collection method because it is well established as a means of simulating social situations in which children portray a range of social roles, including those of adult figures, and articulate socially shared scripts [49,50,51]. At the same time, given longstanding critiques of role-play as a method for depicting children’s actual behavior in real-life interactions [52], data generated through this task were not assumed to represent concrete or accurate parent–child feeding episodes. Rather, the analytic focus was on identifying the range and types of feeding-related strategies that children articulated as part of their internalized understanding of parental discourse.

Two role-play scenarios were developed to reflect common food-related interactions in young children’s everyday lives. The first scenario portrayed a parent attempting to persuade a child to eat a food the child had rejected, while the second depicted a child attempting to convince a parent to permit the consumption of a food the parent disapproved of. In both scenarios, the participating child was instructed to take on the role of the ‘doll-parent,’ thereby expressing internalized parental feeding strategies, while the interviewer enacted the role of the ‘doll-child’.

Within this structured role assignment, children were given substantial autonomy. They selected the gender of both the adult and child dolls and chose the specific foods discussed, ensuring relevance to their own tastes and experiences. They also freely determined the arguments, explanations, and communicative strategies used during the interaction. The interviewer introduced each scenario using a neutral, open-ended prompt tailored to the child’s selected dolls (e.g., “Dad wants her to eat something she doesn’t like to eat. What do you think it could be? What doesn’t she like to eat that Dad thinks she should eat?”). No examples of arguments or strategies were suggested, and the interviewer did not introduce new food items or evaluative language beyond what the child proposed.

To encourage the expression of a range of feeding-related strategies by each participant, the interviewer, in the ‘doll-child’ role, engaged in a brief and mild confrontation, resisting the parent’s suggestions. This involved simple, repetitive child-like responses (e.g., “Please, Daddy,” “I really want it,” or “But I don’t like that”). The resistance was limited to a few short turns and was designed to invite elaboration rather than to challenge or redirect the child’s narrative. Each interaction concluded with acceptance and a positive closing (e.g., “Okay. Thanks for taking care of me Mommy. Hug!”), ensuring that the exchange ended in a supportive tone.

#### 2.4.2. Food-Choice Elicitation Sequence

The second stimulus consisted of a three-part sequence designed to elicit children’s food-choice considerations in a decision-making context. First, children viewed a 48 s animated video developed specifically for the study by the research team. The video depicted nine characters of varying gender, age, and social roles (e.g., parents, a doctor, a police officer, a friend), each recommending the consumption of a different food. All foods featured in the video were selected according to three criteria: (a) they were commonly consumed items in Israeli households and kindergarten settings; (b) they were widely available and culturally typical across the participating communities; and (c) they represented foods that were conventionally categorized as more or less nutritionally recommended according to Israeli Ministry of Health dietary guidelines [53]. Prior to data collection, the food images were informally shown to kindergarten children of the same age group to ensure immediate recognition and familiarity, and all items were readily identified. In addition, the images used in both the video and the memory-matching task were reviewed and approved by experts in early childhood education, educational visualization, and health communication. Each food item was depicted as a clear, standardized image of approximately equal size on a white background with no branding. Visual distractions were intentionally minimized in order to reduce extraneous cognitive load and avoid disrupting children’s attention during the tasks. Each character delivered a brief, simple recommendation consisting of a single sentence (e.g., a father character stating, “You should eat salad,” or a friend character saying, “Grapes are tasty and fun!”), ensuring clarity and developmental appropriateness.

Following the video, children completed a memory-matching game in which they paired images of the characters with images of the foods they had recommended. When necessary, the interviewer replayed the video to support accurate matching, thereby reinforcing children’s awareness of who recommended which food. Although, to the best of our knowledge, no prior study has used an identical memory-matching tool to explore children’s recollection of food recommendations, memory games have been employed in nutrition research with children and considered appropriate for assessing memory and related cognitive outcomes in dietary contexts [54,55].

**Figure 2:**
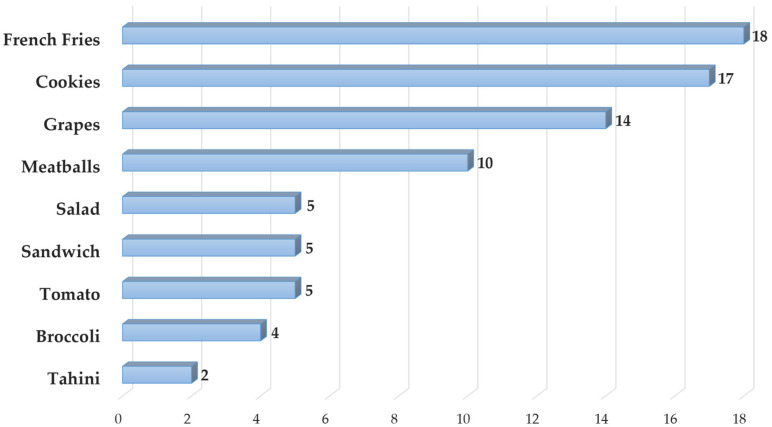
Number of selections for each food item (total selections = 80; two choices per child, *N* = 40).

Immediately after the memory game, while the completed matching board remained visible, children engaged in a simulated grocery shopping task. Simulations of this kind have been used in previous research to study children’s food choices and underlying considerations [56,57,58]. The children were given two ‘bills’ of game money and presented with a mock grocery store consisting of picture cards depicting the nine foods introduced in the video (for a list of the items, see Figure 2). They were asked to “buy” any two food items of their choice. After making their selections, children were invited to explain their choices, and the interviewer encouraged elaboration by asking follow-up questions about the reasons underlying their decisions. The task was intended to elicit children’s expressed reasoning within a structured, simulated context. Although it does not capture real-world purchasing or eating behavior, it offers insight into the range of considerations children may articulate when engaging in food-choice decisions.

### 2.5. Data Analysis

All audio-recorded sessions were transcribed verbatim and checked for accuracy. The transcripts were read repeatedly to achieve close familiarity with the data. Analysis was conducted manually by the first author using reflexive thematic analysis as outlined by Braun and Clarke [59,60], informed by a constructivist epistemological stance.

The two stimuli were initially analyzed separately. Transcripts from the doll role-play task were first examined to identify children’s expressed representations of parental feeding-related strategies, while transcripts from the food-choice simulation were analyzed to capture children’s stated food-choice considerations. Initial coding was conducted in an inductive, data-driven manner, without a predefined framework. Coding considered both semantic (explicit) and latent (interpretive) meanings. A running code list was maintained and iteratively refined; codes were merged, renamed, or collapsed as conceptual overlap emerged.

Analytic memos and informal notes (e.g., “’Want’ and ‘like’ feel overlapping and sometimes appear in the same quote, consider merging”) were maintained throughout this phase to document emerging ideas, questions, and reflexive observations. Codes were then examined for patterns of similarity and difference and iteratively grouped into candidate themes within each stimulus, guided by conceptual coherence rather than frequency. Throughout the coding and theme refinement process, analytic discussions were held iteratively among the authors. These discussions supported critical examination of coding decisions, challenged emerging interpretations, and contributed to the refinement and clarification of thematic structures (see Table 1 for examples of analysis progression).

Following the development of themes within each stimulus, the analyses were brought into dialogue through a comparative analytic phase. Children’s portrayals of parental feeding strategies during the doll role-play were considered alongside their food-choice considerations during the shopping simulation. Particular analytic attention was given to points of convergence as well as to instances of divergence, ambiguity, and mismatch between children’s discursive representations and their enacted choices. This was guided by the assumption that if children’s representations of parental feeding discourse function as directly internalized guides for action, then some degree of convergence would be expected between the strategies attributed to parents in the role-play and the considerations invoked during the shopping simulation. Conversely, divergence between these domains would suggest that children relate to parental discourse selectively and contextually rather than applying it uniformly across situations. The comparison between tasks was therefore designed to explore how children represented parental messages in relation to their own portrayed food-choice reasoning within the structured play contexts, rather than to assess consistency as an indicator of accuracy or internalization. Alignment was operationalized at the level of the individual child using a binary classification (aligned/not aligned), defined as the presence of at least one shared consideration across tasks. A comparison table was created to support this cross-task analysis, and ambiguous cases were resolved through team discussion and re-examination of transcripts. These contrasts were treated as analytically meaningful rather than as inconsistencies and informed the interpretation of children’s agency and contextual variation in food-related meaning-making.

Analysis continued until thematic saturation was judged to have been reached, defined as the stage at which additional transcripts did not generate substantively new or meaningfully distinct themes relevant to the research questions [61].

### 2.6. Ensuring Rigor and Trustworthiness

Rigor in this qualitative study was addressed through attention to credibility, dependability, confirmability, and transferability, consistent with established criteria for trustworthiness in qualitative research [62]. These strategies were integrated throughout the design, data generation, and analytic processes.

Credibility was ensured through the use of complementary tools that allowed children to articulate food-related meanings in different ways. These included discursive representations during role-play and applied decision-making during the shopping simulation. Together, these approaches enriched the data, supported a nuanced understanding of children’s perspectives, and served as a form of cross-task triangulation to strengthen credibility. Furthermore, conducting sessions in familiar settings and monitoring children’s comfort and engagement (e.g., stating there were no “wrong” answers) supported the authenticity of children’s self-expression. Ongoing peer analytic discussions further enhanced the credibility of interpretations. Given the young age of participants and the developmental demands involved in critically evaluating analytic summaries, formal member checking was not conducted.

Dependability was enhanced through a systematic and transparent analytic process. This included the use of verbatim transcriptions and the application of reflexive thematic analysis as described in Section 2.5. The iterative nature of the coding process, supported by regular analytic discussions among the research team, ensured that the thematic structure was stable and consistently applied across the dataset.

Confirmability was addressed through reflexive practices detailed in Section 2.2. This ensured that the findings were grounded in participants’ expressions rather than the researchers’ a priori assumptions about childhood agency.

Conceptual transferability is supported through detailed description of the research context, participants, and methods. While the findings are not intended for statistical generalization, the detailed reporting of participating children’s age range, cultural setting, and educational environment, as well as the specific prompts used, allows readers to assess the relevance of the findings and conceptual applicability to other early childhood health education contexts.

## 3. Results

The findings are presented as follows. First, themes reflecting children’s interpretations of parental feeding strategies during the doll role-play are described. Next, themes identified in children’s considerations during the grocery shopping simulation are presented. Illustrative quotes are presented within the text, with additional examples provided in Table 2. Finally, portrayed parental strategies and stated food-choice considerations are compared to explore patterns of alignment and divergence.

### 3.1. Children’s Expressions of Their Interpretations of Parental Feeding Strategies, Expressed During the Doll Role-Play

**Figure 3:**
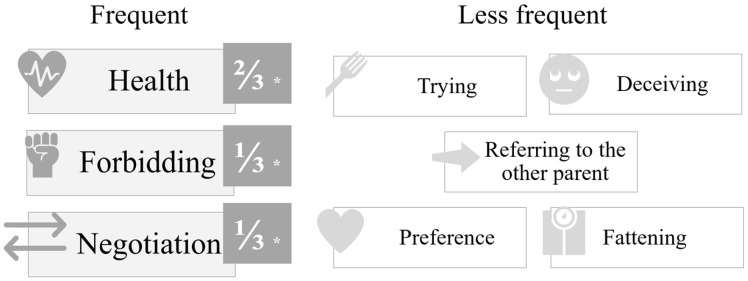
Parental feeding strategies as reflected in children’s expressions during the doll role play.

Children introduced a variety of strategies for influencing the ‘doll-child’s’ food choices while playing the ‘doll-parent’. In the analysis of the presented strategies, three themes were notably frequent (health, control, negotiation) and five less frequent (encouraging to try, deceiving, referring to the other parent, preference, fattening) (see Figure 3).

#### 3.1.1. Frequently Expressed Strategies

Participating children frequently demonstrated feeding strategies that included arguments related to health, controlling behaviors, and negotiations.

Health-related arguments. The most common strategy used by children playing the ‘doll-parent’ consisted of indicating the foods’ health-related attributes, whether positively or negatively (*n* = 27). Children playing the ‘doll-parent’ role tried to convince the ‘doll-child’ to eat certain foods because they would improve their health or facilitate a desired physiological outcome, such as strength or growth: “Eat this [salad] and you’ll be healthy” (boy, 5 years, low-medium SES area); “Eat it [tahini] or you won’t be strong” (girl, 5 years, 5 months, high SES area); “[Eating vegetables] will make you grow” (boy, 5 years, 6 months, high SES area). Health was also the most common argument used when attempting to ward off the ‘doll-child’ from eating undesirable foods. Children playing ‘doll-parents’ claimed that certain foods should not be eaten because they are unhealthy: “It isn’t healthy to eat lots of candy” (girl, 5 years, 5 months, low-medium SES area) or “It [gummy candy] is not healthy for the stomach, for the back, for your whole body.” (boy, 5 years, 8 months, low-medium SES area). Dental hygiene was often specifically mentioned when discussing sweets: “Chocolate is not healthy for your teeth” (boy, 5 years, low-medium SES area), or “It [candy] is too sweet and not healthy for your teeth” (girl, 5 years, 7 months, low-medium SES area).

Control. Behaviors of control, such as denying, coercing, threatening, or limiting, were also frequent among the children’s expressions. About a third of the children (*n* = 13) portrayed ‘doll-parents’ who forbade the ‘doll-children’ to eat undesirable food, stating no reason: “I don’t allow it [pizza]. We don’t eat it at home or in the yard,” (girl, 5 years, high SES area), or just: “No, no, no!” (boy, 5 years, low-medium SES area). They also demonstrated limiting the maximum portions allowed for such foods: “You can only have one or two [cookies]. I won’t give you anymore” (girl, 5 years, 8 months, high SES area). Fewer children playing the ‘doll-parents’ used other controlling behaviors to make the ‘doll-child’ eat desirable foods, with no explanations, like coercing (*n* = 4): “You have to eat it [vegetables]” (girl, 4 years, 11 months, low-medium SES area); threatening (*n* = 5): “Eat it [vegetables] right now, or I will punish you” (boy, 5 years, 8 months, low-medium SES area); or demanding consumption of a minimum amount (*n* = 2): “[Eat] at least one serving of fruit salad” (boy, 5 years, 5 months, low-medium SES area).

Negotiation. About a third of the children, as ‘doll-parents’, applied bargaining behaviors regarding food choices (*n* = 14). Their tactics ranged from promising dessert in exchange for eating the food desired by the ‘doll-parent: “Eat your broccoli, and then you can have a cookie” (boy, 5 years, 6 months, low-medium SES area); to offering a substitute: “If you don’t want a cucumber you can have a carrot” (boy, 5 years, low-medium SES area); or preconditioning the consumption of an undesirable food: “[You can have a lollypop] only if you promise to brush your teeth, and don’t lie!” (boy, 5 years, 8 months, low-medium SES area).

#### 3.1.2. Less Frequent Strategies

Feeding strategies less frequently exhibited by participating children were encouragement to try certain foods (*n* = 8), deception (*n* = 4), referral to the other parent (*n* = 5), stressing preferences (*n* = 5), and advocating avoiding fattening foods (*n* = 3).

Encouragement to try was expressed through urging the ‘doll-child’ to taste the suggested: “Just taste it [vegetable salad]. Just try. Try it again and again and then you’ll see you like it” (boy, 5 years, 1 month, low-medium SES area).

Deception was used by a few children, who misled the ‘doll-child’ by claiming that one food would be served while actually providing another: “[A child explaining about the ‘doll-mother’ she played] she will cut the banana, and the ‘doll-child’ will think it’s marshmallow and eat it” (girl, 5 years, 8 months, high SES area).

Deferring to the other parent appeared when children directed the ‘doll-child’ to the other ‘doll-parent,’ implying that this parent should address the issue: “[about ketchup] Go ask your father” (girl, 4 years, 11 months, low-medium SES area).

Appeals to preference were used when children acting as ‘doll-parents’ emphasized taste or liking, either to encourage consumption: “[regarding vegetables] … but it’s tasty!” (girl, 5 years, high SES area); or to discourage it: “[regarding bread] … But you don’t like it” (boy, 5 years, 2 months, high SES area).

References to fattening outcomes were rare and involved concerns about becoming fat or changes in body appearance as a reason for rejecting: “it [candy] makes you fat” (boy, 5 years, 8 months, low-medium SES area); “do you want kids to call you names [because you would become fat]?” (girl, 5 years, 8 months, high SES area).

### 3.2. Grocery Shopping Simulation—Children’s Food Choices

Food items chosen most frequently by the children were foods considered less healthy, such as French fries and cookies. The least-chosen food item was tahini (for the frequency of selections for each food item, see Figure 2).

#### Expressed Food-Choice Considerations

Two main types of considerations were expressed by the children during the grocery shopping simulation: personal preference and health.

Almost two-thirds of the children’s considerations (26/40) had to do with their preference—choosing a particular food because they liked it or wanted it: “[I chose] cookies and French fries because they’re candy, and I like candy” (girl, 5 years, high SES area); “Broccoli and grapes, because this is what I want” (girl, 5 years, 8 months, high SES area); “because I like grapes, and I want to eat them” (girl, 5 years, 1 month, low-medium SES area). When the children were asked to explain why they did not choose other foods from the offered selection, once again, about two-thirds of their responses involved their preference: [interviewer: “why didn’t you choose the broccoli?”] “Because I don’t like it” (boy, 5 years, low-medium SES area); or “I don’t want [tahini] because I like grapes better” (girl, 5 years, 1 month, low-medium SES area).

About one-third of the children (14/40) expressed health-related considerations. For example, a child choosing salad and meatballs said: “Because they’re the healthiest options” (boy, 5 years, 8 months, low-medium SES area); another, not choosing French fries, claimed, “Because they’re not so healthy” (girl, 5 years, 1 month, low-medium SES area).

None of the children mentioned the video figures’ recommendations as a consideration for choosing foods despite the video having been played and the matching game experienced just prior to the shopping simulation. When asked about it, some children recognized the figures’ advice but chose to disregard it. For example, a boy who chose cookies and grapes “because they’re tasty,” when asked about not choosing tahini, although recommended by the teacher in the video, simply responded, “because I don’t want to” (boy, 5 years, 3 months, high SES area).

### 3.3. Comparing Children’s Expressions Reflecting Parental Feeding-Related Strategies and Children’s Food-Choice Considerations

An examination of the relationship between portrayals of parents’ feeding-related strategies during the doll role-play and children’s food-choice considerations during the shopping task indicated that, in most cases, these did not closely align. While ‘shopping,’ 70% of the children applied considerations different from those stated while playing the ‘doll-parent’ role. Most of them used health arguments in the ’doll-parent’ role and personal preference considerations while ‘shopping’. The remaining children (30%), however, exhibited some congruencies between parental strategies and their food-choice considerations, mentioning health considerations in both the ‘doll-parent’ role and the grocery shopping simulation.

## 4. Discussion

This study examined young children’s expressed representations of parental feeding strategies alongside their own stated food-choice considerations, with the aim of bringing attention to children’s perspectives within food-related socialization processes. Our findings suggest that health-related considerations are highly salient in children’s representations of how parents frame food-related decisions, as reflected in the frequent use of health arguments during the doll role-play. Children commonly portrayed parents as emphasizing health when encouraging or discouraging the consumption of particular foods. However, this salience in children’s representations did not translate directly into their own food-choice considerations expressed during the shopping simulation, which more often represented personal preferences.

This divergence between children’s portrayals of parental discourse and their own stated considerations highlights the complexity of food-related meaning-making in early childhood. While parents may be perceived as bringing attention to health as an important consideration, children’s immediate choices appeared to reflect a different set of priorities. This pattern does not necessarily indicate a lack of awareness of health-related messages. Notably, all children successfully completed the memory-matching task, accurately identifying which authority figures recommended each food item. Thus, the absence of references to these recommendations during the shopping simulation cannot be attributed to failed recall. At the same time, successful recall does not preclude the possibility that the video shaped how health-related categories were framed or activated within the interaction. Exposure to recommendations from socially salient figures may have heightened awareness of health-related categories and activated a more normative discourse frame. The role-play setting, conducted in a school environment and in interaction with an adult researcher, may have further encouraged the expression of socially desirable “health talk,” potentially foregrounding normative considerations over hedonic preferences. Yet these recommendations were not invoked when children explained their selections. This suggests that the video stimulus may have influenced attention and memory without directly determining choice behavior. Rather than reflecting misunderstanding, the findings point to children’s capacity to distinguish between recognizing authoritative advice and exercising personal preference in contexts perceived as affording autonomy. Such differentiation is consistent with research conceptualizing children as active agents in their own socialization processes [35,36].

Research on kindergarten children’s food-related knowledge further suggests that young children construct food concepts from multiple epistemic sources, including personal experience, environmental exposure, and perceived adult messages [44]. In that work, “health” frequently emerged as a category rooted in perceived messages rather than embodied experience, indicating that health-related discourse may function as socially transmitted evaluative knowledge. This distinction may help explain the present divergence: children may readily reproduce health-oriented language in normative contexts such as role-play, while drawing more heavily on preference-based, experiential knowledge when articulating their own selections. Broader reviews of food literacy development similarly emphasize that the ability to integrate abstract nutritional concepts into decision-making evolves with cognitive maturity and that younger children often rely more strongly on immediate sensory and affective cues than on long-term health [63]. From a developmental perspective, therefore, the observed divergence may reflect not inconsistency, but the coexistence of emerging conceptual knowledge and developmentally salient motivational factors.

Importantly, parents’ emphasis on health as a food-choice consideration may serve functions beyond shaping children’s immediate eating decisions. By repeatedly stressing health, parents may convey to children what constitutes a desirable way of eating and establish normative rules that guide understanding of appropriate food behavior over time. Through such messages, children acquire knowledge about nutritious foods and health-related values, which they may draw upon later in life, when health considerations often become more prominent in food-choice decision-making [64,65,66]. From this perspective, the presence of health discourse in children’s role-play expressions can be understood as part of a longer-term socialization process, even when health does not feature centrally in children’s current choices.

The prominence of foods such as French fries and cookies in children’s selections warrants careful interpretation. Although child-targeted food advertising has been shown to influence children’s food preferences and requests [24,25], the present findings should not be interpreted as evidence of advertising effects alone. Foods such as these are not only heavily marketed but also highly familiar, frequently consumed, and commonly modeled by adults, siblings, and peers. Prior research indicates that repeated exposure, social modeling, and shared consumption practices play a substantial role in shaping children’s preferences [67,68]. In addition to these social and environmental influences, biological taste preferences may also contribute. Research has shown that young children naturally prefer higher levels of sweet and salty tastes, reflecting basic sensory predispositions that are evident in early development and can make energy-dense, sweet, and salty foods particularly appealing to this age group [69]. Thus, children’s frequent selection of these foods likely reflects the convergence of multiple social, experiential, and biologically rooted influences.

Children’s portrayals of parental feeding strategies further illuminate their understanding of adult authority and influence. Expressions of controlling strategies (e.g., denying access, coercion, or threats) and manipulative practices (e.g., deception or referral to the other parent) indicate children’s awareness of such approaches but do not allow conclusions about their effectiveness in shaping children’s actual eating behavior. The findings indicate that children recognize these practices as part of parental food-related discourse while simultaneously exercising agency in their own food-choice reasoning. This interpretation aligns with existing evidence showing that controlling or manipulative feeding practices may be associated with resistance, conflict, or reduced self-regulation rather than compliance [30,70].

Negotiation strategies were also frequently portrayed, a finding that merits cultural consideration. The relatively high frequency of negotiation-based portrayals may reflect broader communicative norms within Israeli non-Orthodox Jewish families, which are characterized by relatively low power distance and a tendency to involve children in family decision-making [71]. In such contexts, parents may be perceived as seeking compromise rather than unilateral control. The confidence with which children enacted food choices during the shopping simulation, even when contradicting advice from authoritative adult figures in the video, further suggests that children perceived food choice as a legitimate domain for exercising discretion. Future research could examine whether similar patterns emerge in cultural contexts characterized by different norms regarding authority, obedience, and children’s participation in decision-making. For example, in some cultures, such as Japan, young children are commonly expected to eat the foods they are served regardless of personal preferences, and negotiation around food may be less emphasized [72]. Comparative studies across cultural settings could help clarify how cultural norms shape children’s understandings of parental feeding practices and their own food-related agency.

The limited appearance of fattening-related arguments, particularly those referencing body appearance, is noteworthy. Studies suggest that children’s talk about body size often includes social meanings (e.g., teasing, attractiveness, “fitting in”), but the visibility and directness of these themes can vary by age, context, and how questions are elicited [73]. At the same time, research suggests that elements of weight bias and thin-ideal valuation can be present from the preschool years, even when children do not consistently articulate these ideas explicitly [74,75]. Against this background, children’s relative avoidance of explicit references to fatness in the current study may reflect broader norms that discourage overt stigmatizing talk, with “health” functioning as a more socially acceptable frame for discussing food and bodies [76,77]. Nevertheless, one child’s reference to social ridicule is consistent with evidence that even young children can link larger body size with negative social evaluations [78,79].

Prior work also suggests that gendered patterns can emerge early, with some studies finding stronger appearance or weight-related biases among girls, while also showing that boys may endorse similar stereotypes and preferences in some contexts [74]. In this light, the fact that only a few children raised appearance-related themes, and that these children were not exclusively girls, may reflect heterogeneity in children’s salience of body-related meanings, differential exposure to appearance ideals, and/or variation in comfort with making such themes explicit in a research setting [74,80]. Overall, these findings suggest that concerns about weight and appearance may be present but expressed indirectly or selectively, underscoring the need for further research into how young children interpret and internalize obesity- and appearance-related discourse [73,75].

Taken together, the findings invite reflection on their possible implications for health promotion and nutrition education. Although the present study did not evaluate interventions directly, it suggests that young children engage with food-related messages selectively and in context-dependent ways. Health-related arguments were prominent in children’s portrayals of parental discourse, yet their own choices were more often guided by personal preference. This pattern may indicate that educational efforts relying exclusively on adult-framed health messaging do not always align with how children experience everyday food decisions. Interventions might therefore benefit from incorporating structured opportunities for children to express their own reasoning, preferences, and interpretations, rather than positioning them solely as recipients of guidance.

More broadly, these findings resonate with participatory approaches in child health research that emphasize children’s right to be heard in matters affecting their well-being [6,81]. Engaging children’s voices in the development of nutrition-related initiatives may enhance the contextual relevance of messaging and help bridge gaps between expert recommendations and lived experience [82]. Participatory processes have been associated with improved alignment between interventions and everyday realities and may also support children’s sense of agency and health literacy [81,83]. While further empirical work is needed to examine how such approaches operate in early childhood contexts, the present study underscores the potential value of treating children’s expressed perspectives as a meaningful resource in the design of responsive and context-sensitive health education efforts.

This study has several limitations that should be considered when interpreting the findings. First, the sample comprised 40 kindergarten children from a specific cultural and geographic context in northern Israel. Although qualitative research does not aim for statistical generalization, the sociocultural norms surrounding parenting, authority, and food practices may shape children’s representations in ways that differ in other settings.

Second, the findings are based on children’s verbal expressions within structured, play-based research tasks. While doll role-play and simulated shopping were selected as developmentally appropriate elicitation tools, they do not directly capture children’s behavior in naturalistic eating situations. Children’s responses may have been influenced by the research setting, their interaction with an adult researcher, or their interpretations of the task demands.

Finally, the study design captures children’s perspectives at a single point in time. Longitudinal research could further clarify how children’s representations of parental feeding discourse and their own food-choice reasoning evolve across developmental stages [39].

## 5. Conclusions

This study contributes to ongoing efforts to foreground children’s perspectives within research on food socialization and healthcare and health education. By examining young children’s representations of parental feeding strategies alongside their own stated food-choice considerations, the findings highlight the interpretive and context-sensitive nature of early food-related meaning-making. The observed divergence between parental discourse as portrayed by children and their own expressed decision-making underscores the importance of approaching children not merely as recipients of guidance, but as active participants in food-related interactions. Continued research across diverse cultural and developmental contexts may further clarify how children’s voices can inform responsive and contextually grounded health promotion efforts.

## Figures and Tables

**Table 1:** Illustrative examples of progression from raw excerpts to final themes.

**Doll role-play:**			
**Raw Excerpt**	**Initial Code**	**Candidate theme**	**Final Theme**
“You know what? I’ll make you an egg, and then you can have dessert.”	Dessert at the End	Rewards	Negotiation
“You need to eat this so you can grow.”	To Help You Grow	Positive body development	Health
“No, sweetie, you can only have one.”	Portion Limitation	Boundaries	Control
**Simulated shopping:**			
**Raw Excerpt**	**Initial Codes**	**Candidate theme**	**Final Theme**
“Because I like it.”/“I want it.”	Liking; Desire	Preference	Personal Preference

**Table 2:** Thematic Categories and Exemplar Quotes from Both Tasks.

**Doll role-play**	
**Illustrative Quote**	**Theme**
**“If you don’t eat this, you can’t go to kindergarten because you’ll get sick.”**	Health
**“Eat, or I’ll take your game away.”**	Control
**“If you’ll eat dinner I’ll make you a pancake.”**	Negotiation
**“Just try it first.”**	Encouraging to try
**“I’ll put snacks in the salad so he can’t see the vegetables, and then he’ll eat it.”**	Deceiving
**“Ask Mommy.”**	Referring to the other parent
**“But you don’t like this black thing.”**	Preferences
**“Because you’re very fat.”**	Fattening
**Shopping simulation**	
**Illustrative Quote**	**Theme**
**“Because it’s candy, and I like candy.”**	Personal preference
**“I like it, but it’s not healthy, so I don’t eat a lot of it.”**	Health

## Data Availability

The raw de-identified data supporting the conclusions of this article will be made available by the authors on request.

## References

[1] Horesh A., Tsur A.M., Bardugo A., Twig G. (2021). Adolescent and Childhood Obesity and Excess Morbidity and Mortality in Young Adulthood—A Systematic Review. Curr. Obes. Rep..

[2] Lobstein T., Brinsden H. (2019). The Childhood Obesity Atlas.

[3] Spinelli A., Buoncristiano M., Nardone P., Starc G., Hejgaard T., Júlíusson P.B., Fismen A.S., Weghuber D., Musić Milanović S., García-Solano M. (2021). Thinness, Overweight, and Obesity in 6- to 9-Year-Old Children from 36 Countries: The World Health Organization European Childhood Obesity Surveillance Initiative—COSI 2015–2017. Obes. Rev..

[4] World Health Organization (2006). Global Strategy on Diet, Physical Activity and Health: A Framework to Monitor and Evaluate Implementation.

[5] Wright-Pedersen S., Vidgen H., Gallegos D. (2024). Children’s Descriptions of Their Involvement within Everyday Food Practices. Appetite.

[6] Quigley F., Lynch L., Price R., Hollywood L., Gallagher A.M., Mooney E., McCloat A., Moorhead S.A. (2025). ‘I Want Food to Be Tasty and Healthy’: School-Children’s Experiences with Nutrition Education and Messaging. Health Promot. Int..

[7] Young S.L. (2022). The Value of Children’s Voices in Public Health Research. J. Nutr..

[8] Clark H.R., Goyder E., Bissell P., Blank L., Peters J. (2007). How Do Parents’ Child-Feeding Behaviours Influence Child Weight? Implications for Childhood Obesity Policy. J. Public Health.

[9] Daniels L.A. (2019). Feeding Practices and Parenting: A Pathway to Child Health and Family Happiness. Ann. Nutr. Metab..

[10] Birch L.L., Fisher J.O., Grimm-Thomas K., Markey C.N., Sawyer R., Johnson S.L. (2001). Confirmatory Factor Analysis of the Child Feeding Questionnaire: A Measure of Parental Attitudes, Beliefs and Practices about Child Feeding and Obesity Proneness. Appetite.

[11] Chawner L.R., Blundell-Birtill P., Hetherington M.M. (2023). Parental Intentions to Implement Vegetable Feeding Strategies at Home: A Cross Sectional Study. Appetite.

[12] Hughes S.O., Power T.G., Orlet Fisher J., Mueller S., Nicklas T.A. (2005). Revisiting a Neglected Construct: Parenting Styles in a Child-Feeding Context. Appetite.

[13] Sandvik P., Kuronen S., Reijs Richards H., Eli K., Ek A., Somaraki M., Nowicka P. (2022). Associations of Preschoolers’ Dietary Patterns with Eating Behaviors and Parental Feeding Practices at a 12-Month Follow-up of Obesity Treatment. Appetite.

[14] Vollmer R.L., Mobley A.R. (2013). Parenting Styles, Feeding Styles, and Their Influence on Child Obesogenic Behaviors and Body Weight. A Review. Appetite.

[15] Bova A., Arcidiacono F. (2014). “You Must Eat the Salad Because It Is Nutritious”. Argumentative Strategies Adopted by Parents and Children in Food-Related Discussions at Mealtimes. Appetite.

[16] Zeinstra G.G., Koelen M.A., Kok F.J., De Graaf C. (2007). Cognitive Development and Children’s Perceptions of Fruit and Vegetables; a Qualitative Study. Int. J. Behav. Nutr. Phys. Act..

[17] Corsini N., Danthiir V., Kettler L., Wilson C. (2008). Factor Structure and Psychometric Properties of the Child Feeding Questionnaire in Australian Preschool Children. Appetite.

[18] Russell C.G., Worsley A., Campbell K.J. (2015). Strategies Used by Parents to Influence Their Children’s Food Preferences. Appetite.

[19] Loth K.A., Uy M., Neumark-Sztainer D., Fisher J.O., Berge J.M. (2018). A Qualitative Exploration into Momentary Impacts on Food Parenting Practices among Parents of Pre-School Aged Children. Appetite.

[20] Berge J.M., Trofholz A., Schulte A., Conger K., Neumark-Sztainer D. (2016). A Qualitative Investigation of Parents’ Perspectives About Feeding Practices With Siblings Among Racially/Ethnically and Socioeconomically Diverse Households. J. Nutr. Educ. Behav..

[21] Molina P., Coloma M.J., Gálvez P., Stecher M.J., Vizcarra M., Schwingel A. (2023). Food Parenting Practices Promoted by Childcare and Primary Healthcare Centers in Chile: What Influences Do These Practices Have on Parents? A Qualitative Study. Children.

[22] Bronfenbrenner U. (1992). Ecological Systems Theory.

[23] Darling N. (2007). Ecological Systems Theory: The Person in the Center of the Circles. Res. Hum. Dev..

[24] Boyland E.J., Whalen R. (2015). Food Advertising to Children and Its Effects on Diet: Review of Recent Prevalence and Impact Data. Pediatr. Diabetes.

[25] Folkvord F., Anschütz D.J., Boyland E., Kelly B., Buijzen M. (2016). Food Advertising and Eating Behavior in Children. Curr. Opin. Behav. Sci..

[26] Faith M.S., Tepper B.J., Hoffman D.J., Pietrobelli A. (2002). Genetic and Environmental Influences on Childhood Obesity. Clin. Fam. Pract..

[27] Heller R.L., Chiero J.D., Trout N., Mobley A.R. (2021). A Qualitative Study of Providers’ Perceptions of Parental Feeding Practices of Infants and Toddlers to Prevent Childhood Obesity. BMC Public Health.

[28] Brown R., Ogden J. (2004). Children’s Eating Attitudes and Behaviour: A Study of the Modelling and Control Theories of Parental Influence. Health Educ. Res..

[29] Pace U., D’Urso G., Zappulla C. (2018). Negative Eating Attitudes and Behaviors among Adolescents: The Role of Parental Control and Perceived Peer Support. Appetite.

[30] Wardle J., Carnell S., Cooke L. (2005). Parental Control over Feeding and Children’s Fruit and Vegetable Intake: How Are They Related?. J. Am. Diet. Assoc..

[31] Wright-Pedersen S., Vidgen H., Gallegos D. (2026). Children’s Perspectives of Their Everyday Food Practices: Insights to Inform Policy and Interventions. PLoS ONE.

[32] Ndiaye K., Silk K.J., Anderson J., Horstman H.K., Carpenter A., Hurley A., Proulx J. (2013). Using an Ecological Framework to Understand Parent–Child Communication about Nutritional Decision-Making and Behavior. J. Appl. Commun. Res..

[33] Chan M.J., Tay G.W.N., Kembhavi G., Lim J., Rebello S.A., Ng H., Lin C., Wang M.C., Müller-Riemenschneider F., Chong M.F.-F. (2022). Understanding Children’s Perspectives of the Influences on Their Dietary Behaviours. Public Health Nutr..

[34] Oudat Q., Miller E.L., Couch S.C., Lee R.C., Bakas T. (2025). Understanding Caregivers’ Influence on Preschoolers’ Eating Behaviors: An Integrative Review Guided by the Theory of Planned Behavior. Children.

[35] Pontecorvo C., Fasulo A., Sterponi L. (2001). Mutual Apprentices: The Making of Parenthood and Childhood in Family Dinner Conversations. Hum. Dev..

[36] Zotevska E., Martín-Bylund A. (2022). How to Do Things with Food: The Rules and Roles of Mealtime “things” in Everyday Family Dinners. Child. Soc..

[37] Alm S., Olsen S.O., Honkanen P. (2015). The Role of Family Communication and Parents’ Feeding Practices in Children’s Food Preferences. Appetite.

[38] O’Connell R., Brannen J. (2014). Children’s Food, Power and Control: Negotiations in Families with Younger Children in England. Childhood.

[39] Ogden J., Roy-Stanley C. (2020). How Do Children Make Food Choices? Using a Think-Aloud Method to Explore the Role of Internal and External Factors on Eating Behaviour. Appetite.

[40] Gram M. (2015). Buying Food for the Family: Negotiations in Parent/Child Supermarket Shopping. J. Contemp. Ethnogr..

[41] Reilly J.J., Kelly J. (2011). Long-Term Impact of Overweight and Obesity in Childhood and Adolescence on Morbidity and Premature Mortality in Adulthood: Systematic Review. Int. J. Obes..

[42] Schultz C.M., Danford C.M. (2016). Children’s Knowledge of Eating: An Integrative Review of the Literature. Appetite.

[43] Sudarsan I., Hoare K., Sheridan N., Roberts J. (2022). Giving Voice to Children in Research: The Power of Child-centered Constructivist Grounded Theory Methodology. Res. Nurs. Health.

[44] Freedman I., Eilam B., Gesser-Edelsburg A. (2021). Young Children’s Food-Related Knowledge: Kindergarteners’ Free Categorization of Food Items. J. Nutr. Educ. Behav..

[45] United Nations Convention on the Rights of the Child (1989). General Assembly Resolution 44/25.

[46] Olmos-Vega F.M., Stalmeijer R.E., Varpio L., Kahlke R. (2023). A Practical Guide to Reflexivity in Qualitative Research: AMEE Guide No. 149. Med. Teach..

[47] Thomas L. (2024). The Researcher’s Gaze. Doing Good Qualitative Research.

[48] Sibbald K.R., Phelan S.K., Beagan B.L., Pride T.M. (2025). Positioning Positionality and Reflecting on Reflexivity: Moving From Performance to Practice. Qual. Health Res..

[49] O’sullivan C., Cohen L., Manion L., Morrison K. (2007). Role-Playing. Research Methods in Education.

[50] Sutherland L.A., Beavers D.P., Kupper L.L., Bernhardt A.M., Heatherton T., Dalton M.A. (2008). Like Parent, like Child: Child Food and Beverage Choices during Role Playing. Arch. Pediatr. Adolesc. Med..

[51] Dalton M.A., Bernhardt A.M., Gibson J.J., Sargent J.D., Beach M.L., Adachi-Mejia A.M., Titus-Ernstoff L.T., Heatherton T.F. (2005). Use of Cigarettes and Alcohol by Preschoolers While Role-Playing as Adults:“Honey, Have Some Smokes”. Arch. Pediatr. Adolesc. Med..

[52] Van Hasselt V.B., Hersen M., Bellack A.S. (1981). The Validity of Role Play Tests for Assessing Social Skills in Children. Behav. Ther..

[53] The National Program for Active and Healthy Living EfshariBari: Eat Healthy. https://efsharibari.health.gov.il/en/eat-healthy/.

[54] Folkvord F., Laguna-Camacho A. (2019). The Effect of a Memory-Game with Images of Vegetables on Children’s Vegetable Intake: An Experimental Study. Appetite.

[55] Folkvord F., Anastasiadou D.T., Anschütz D. (2017). Memorizing Fruit: The Effect of a Fruit Memory-Game on Children’s Fruit Intake. Prev. Med. Rep..

[56] Carruth B.R., Skinner J.D., Moran J.D., Coletta F. (2000). Preschoolers’ Food Product Choices at a Simulated Point of Purchase and Mothers’ Consumer Practices. J. Nutr. Educ..

[57] Contento I.R., Williams S.S., Michela J.L., Franklin A.B. (2006). Understanding the Food Choice Process of Adolescents in the Context of Family and Friends. J. Adolesc. Health.

[58] Mau G., Schuhen M., Steinmann S., Schramm-Klein H. (2016). How Children Make Purchase Decisions: Behaviour of the Cued Processors. Young Consum..

[59] Braun V., Clarke V. (2006). Using Thematic Analysis in Psychology. Qual. Res. Psychol..

[60] Braun V., Clarke V. (2019). Reflecting on Reflexive Thematic Analysis. Qual. Res. Sport Exerc. Health.

[61] Hennink M., Kaiser B.N. (2022). Sample Sizes for Saturation in Qualitative Research: A Systematic Review of Empirical Tests. Soc. Sci. Med..

[62] Ahmed S.K. (2024). The Pillars of Trustworthiness in Qualitative Research. J. Med. Surg. Public Health.

[63] Ares G., De Rosso S., Mueller C., Philippe K., Pickard A., Nicklaus S., van Kleef E., Varela P. (2024). Development of Food Literacy in Children and Adolescents: Implications for the Design of Strategies to Promote Healthier and More Sustainable Diets. Nutr. Rev..

[64] Steptoe A., Pollard T.M., Wardle J. (1995). Development of a Measure of the Motives Underlying the Selection of Food: The Food Choice Questionnaire. Appetite.

[65] Wardle J., Haase A.M., Steptoe A., Nillapun M., Jonwutiwes K., Bellisie F. (2004). Gender Differences in Food Choice: The Contribution of Health Beliefs and Dieting. Ann. Behav. Med..

[66] Chen P.J., Antonelli M. (2020). Conceptual Models of Food Choice: Influential Factors Related to Foods, Individual Differences, and Society. Foods.

[67] Kral T.V.E., Rauh E.M. (2010). Eating Behaviors of Children in the Context of Their Family Environment. Physiol. Behav..

[68] Suwalska J., Bogdański P. (2021). Social Modeling and Eating Behavior—A Narrative Review. Nutrients.

[69] Mennella J.A. (2014). Ontogeny of Taste Preferences: Basic Biology and Implications for Health. Am. J. Clin. Nutr..

[70] Peters J., Parletta N., Lynch J., Campbell K. (2014). A Comparison of Parental Views of Their Pre-School Children’s ‘Healthy’ versus ‘Unhealthy’ Diets. A Qualitative Study. Appetite.

[71] Hofstede G., Hofstede G.J. (2005). Cultures and Organizations, Software of the Mind: Intercultural Cooperation and Its Importance for Survival.

[72] Freedman I. (2016). Cultural Specificity in Food Choice—The Case of Ethnography in Japan. Appetite.

[73] Lorenc T., Burchett H., Mendizabal-Espinosa R., Stansfield C., Sutcliffe K., Sowden A. (2026). Children’s Views of Obesity, Body Size and Weight: Systematic Review of UK Qualitative Evidence. J. Epidemiol. Community Health.

[74] Tatangelo G., McCabe M., Mellor D., Mealey A. (2016). A Systematic Review of Body Dissatisfaction and Sociocultural Messages Related to the Body among Preschool Children. Body Image.

[75] Paxton S.J., Damiano S.R. (2017). The Development of Body Image and Weight Bias in Childhood. Adv. Child Dev. Behav..

[76] Puhl R., Brownell K.D. (2001). Bias, Discrimination, and Obesity. Obesity.

[77] Lydecker J.A., O’Brien E., Grilo C.M. (2018). Parents Have Both Implicit and Explicit Biases Against Children with Obesity. J. Behav. Med..

[78] Holub S.C. (2008). Individual Differences in the Anti-Fat Attitudes of Preschool-Children: The Importance of Perceived Body Size. Body Image.

[79] Damiano S.R., Gregg K.J., Spiel E.C., McLean S.A., Wertheim E.H., Paxton S.J. (2015). Relationships between Body Size Attitudes and Body Image of 4-Year-Old Boys and Girls, and Attitudes of Their Fathers and Mothers. J. Eat. Disord..

[80] Harriger J., Trammell J., Wick M., Luedke M. (2019). Gender and Age Differences in Pre-schoolers’ Weight Bias Beliefs and Behavioural Intentions. Br. J. Dev. Psychol..

[81] Freire K., Pope R., Jeffrey K., Andrews K., Nott M., Bowman T. (2022). Engaging with Children and Adolescents: A Systematic Review of Participatory Methods and Approaches in Research Informing the Development of Health Resources and Interventions. Adolesc. Res. Rev..

[82] Jilani H., Schilling I., Gerhardus A., Klink U. (2025). Active Involvement of Children Aged 11–12 Years in the Development of a Healthy Nutrition Intervention—A Qualitative Evaluation from Researchers’ and Children’s Perspectives. BMC Public Health.

[83] Scholtes-Bos W., van Lieshout M., van Roost M.H.I., de Vries S.I. (2025). Beyond Healthy Eating: The Broader Impact of the Food Boost Challenge’s Participatory Approach with Young People. Soc. Sci..

